# Global characteristics and trends in research on *Candida auris*

**DOI:** 10.3389/fmicb.2023.1287003

**Published:** 2023-12-06

**Authors:** Qihui Wang, Shitong Cheng, Yinling Wang, Fushun Li, Jingjing Chen, Wei Du, Hui Kang, Zhongqing Wang

**Affiliations:** ^1^Laboratory of Microbiology, Department of Clinical Laboratory, The First Hospital of China Medical University, Shenyang, Liaoning, China; ^2^National Clinical Research Center for Laboratory Medicine, Department of Clinical Laboratory, The First Hospital of China Medical University, Shenyang, Liaoning, China; ^3^Department of Information Centre, The First Hospital of China Medical University, Shenyang, Liaoning, China

**Keywords:** *Candida auris*, nosocomial outbreaks, drug resistance, bibliometric analysis, research trends

## Abstract

**Introduction:**

*Candida auris*, a fungal pathogen first reported in 2009, has shown strong resistance to azole antifungal drugs and has caused severe nosocomial outbreaks. It can also form biofilms, which can colonize patients’ skin and transmit to others. Despite numerous reports of *C. auris* isolation in various countries, many studies have reported contradictory results.

**Method:**

A bibliometric analysis was conducted using VOSviewer to summarize research trends and provide guidance for future research on controlling *C. auris* infection. The analysis revealed that the United States and the US CDC were the most influential countries and research institutions, respectively. For the researchers, Jacques F. Meis published the highest amount of related articles, and Anastasia P. Litvintseva’s articles with the highest average citation rate. The most cited publications focused on clade classification, accurate identification technologies, nosocomial outbreaks, drug resistance, and biofilm formation. Keyword co-occurrence analysis revealed that the top five highest frequencies were for ‘drug resistance,’ ‘antifungal susceptibility test,’ ‘infection,’ ‘*Candida auris,*’ and ‘identification.’ The high-frequency keywords clustered into four groups: rapid and precise identification, drug resistance research, pathogenicity, and nosocomial transmission epidemiology studies. These clusters represent different study fields and current research hotspots of *C. auris*.

**Conclusion:**

The bibliometric analysis identified the most influential country, research institution, and researcher, indicating current research trends and hotspots for controlling *C. auris.*

## 1 Introduction

*Candida auris* was first reported in 2009 and was isolated from the external auditory canal of a Japanese patient (Satoh et al., 2009). Subsequently, there have been a number of reports of *C. auris* being isolated in several other countries, and a retrospective analysis of preserved strains confirmed that *C. auris* existed in many countries before 2009; however, its presence was not discovered due to challenges associated with its biological identification (Kim et al., 2009; Shin et al., 2012; Desnos-Ollivier et al., 2021). Owing to its resistance to azole antifungal drugs and its tendency to cause outbreaks in hospitals, it has attracted considerable attention from the medical community and has become a major threat to public health (Lockhart et al., 2017).

The rapid and accurate identification of *C. auris* is essential for controlling nosocomial outbreaks. At present, traditional identification methods can easily be used to identify *C. haemulonii*, *C. glabrata* and other *Candida* species (Parra-Giraldo et al., 2018). *Candida auris* is usually resistant to fluconazole, and certain clades of *C. auris* exhibit a higher MIC against azole antifungals than do other *Candida* species (Chowdhary et al., 2017). Consequently, the mistaken identification of *C. auris* not only delays patient treatment but also the optimal timing of nosocomial infection control strategies, resulting in nosocomial outbreaks of *C. auris*. With the application of new technologies, such as matrix-assisted laser desorption ionization-time of flight mass spectrometry (MALDI-TOF MS), internally transcribed spacer (ITS) sequencing, and other molecular technologies (Borman et al., 2017), the success rate of *C. auris* identification has also increased.

Studies have shown that *C. auris* quickly adapts to azoles, as well as other antifungal drugs, and increases its MIC against these drugs (Bravo Ruiz et al., 2019; Carolus et al., 2021), which complicates the treatment of infectious diseases caused by *C. auris*. In addition, *C. auris* is able to form biofilms (Eix et al., 2022). Although *C. auris* grows relatively slowly in Yeast Extract Peptone Dextrose (YEPD or YPD) medium, it can quickly form a dense biofilm on irregular folds of human skin without breaking through the skin tissue, which explains its continuous colonization of patients’ skin and its transmission among patients and between patients and the environment. *C. auris* does not have as strong of an ability to produce hyphae as does *C. albicans*; however, it still exhibits considerable virulence (Yue et al., 2018), with different virulence levels exhibited by different strains (Carvajal et al., 2021). Since its discovery, *C. auris* has become one of the main fungal pathogens due to its high drug resistance, difficulty to treat, and its ability to cause large-scale outbreaks.

Although there are many studies on *C. auris*, most are based on a small number of strains, and many of these studies have reported contradictory results. Therefore, caution should be exercised when interpreting these data and results, and more in-depth hotspot-based research with a larger number of strains should be conducted to address the challenges associated with *C. auris*. Bibliometric applies mathematical and statistical methods to analyze changes in the number, distribution, and patterns of published article to describe, compare, and evaluate the hotspots, trends, and contributions of scholars, journals, and countries. To the best of our knowledge, no bibliometric analysis has been conducted on the quantity and quality of *C. auris* research. Therefore, there is an urgent need to summarize the current research status of *C. auris* and to help predict promising keywords and trends. This study aimed to evaluate the research status and trends in *C. auris* to help researchers discover research hotspots in this field and provide a reference for further research.

## 2 Materials and methods

### 2.1 Source database and search strategy

In this study, all article data on *C. auris* was retrieved from the Web of Science (WoS) Core Collection. As the largest comprehensive academic information resource, Web of Science has been utilized as a great tool prior bibliometric studies (Waqas et al., 2020; Ahmad et al., 2021; Saheb et al., 2021; Lin et al., 2022), we chose to use WOS as the data source in this study. The WoS Core Collection database is updated continuously, and it assesses all publications dynamically and rigorously; therefore, it was used for the bibliometric analysis. A WoS-based article analysis was conducted to obtain general information on year of publication, journal, organization, author, and research area distribution. An article search was conducted on the 28 of March 2023. All bibliometric data were downloaded on 28 March 2023 to avoid the impact of database updates.Concerning data collection, the following search strategy was developed: Topic = (‘*Candida auris*’). The language of publication was set to ‘English’. The document type was set to ‘Article’; and the timespan was not set. In total, 750 studies were identified using these search criteria. The ‘Full Record and Cited References’ for the 750 publications were downloaded in ‘Plain Text’ format. As a result, 750 publications were included in the final dataset.

### 2.2 Software application and visualization analysis

VOSviewer is a software tool designed to analyze academic records and create maps based on network data. In this study, all valid data downloaded from the WoSCC database were imported into VOSviewer [version 1.6.19] for visualization analysis, including co-authorship analysis of institutions and authors, co-citation analysis of references, and co-occurrence analysis of keywords. In order to avoid any biases, there were two researchers independently proceed bibliometric strategies. Through the comparison of outcomes, these two independent collected data sets displayed highly consistency.

## 3 Results

### 3.1 Analysis of publications and citation counts

**Figure 1:**
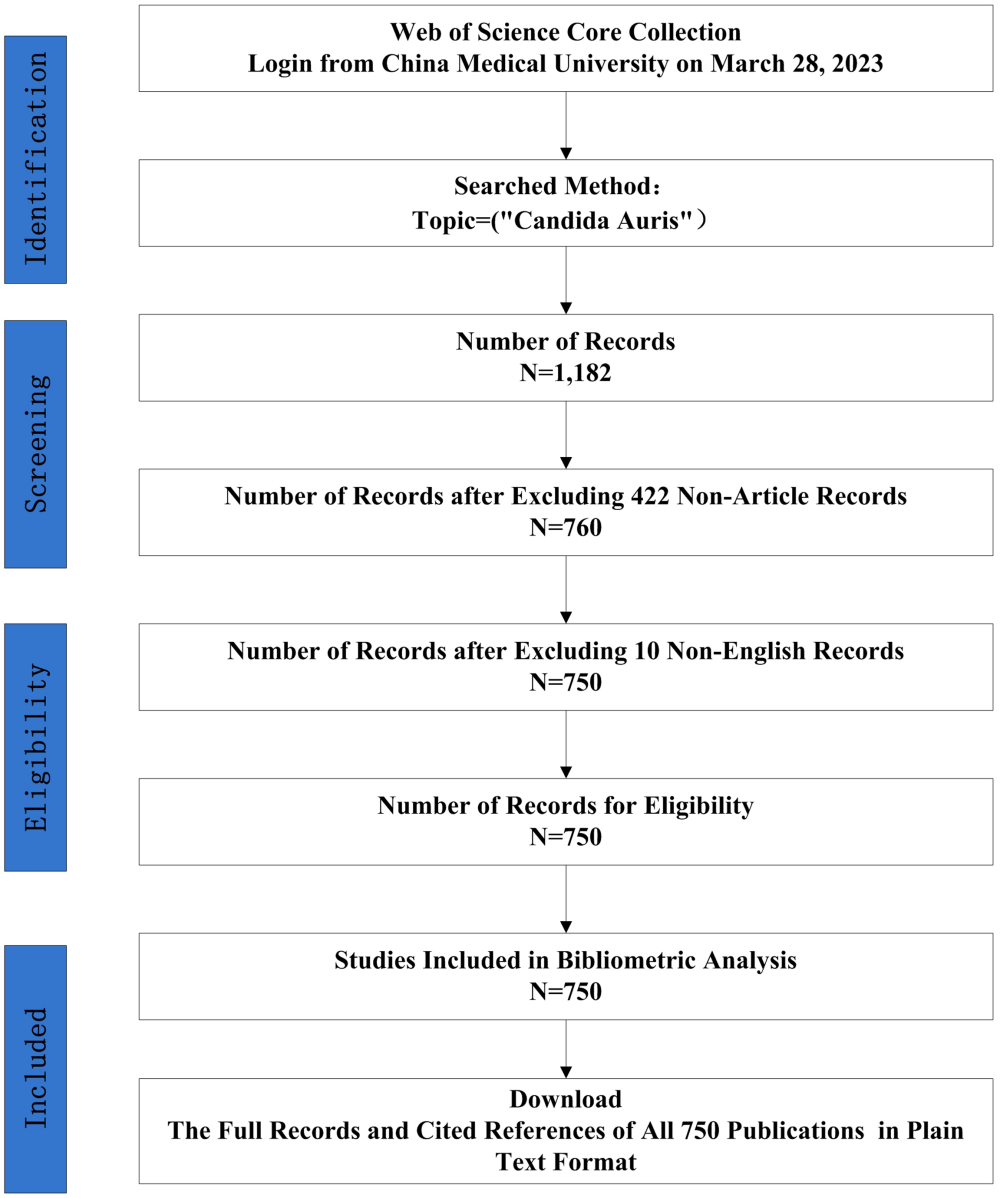
Flow charts of search strategies and article selection methods, followed the PRISMA-ScR guidelines.

**Figure 2:**
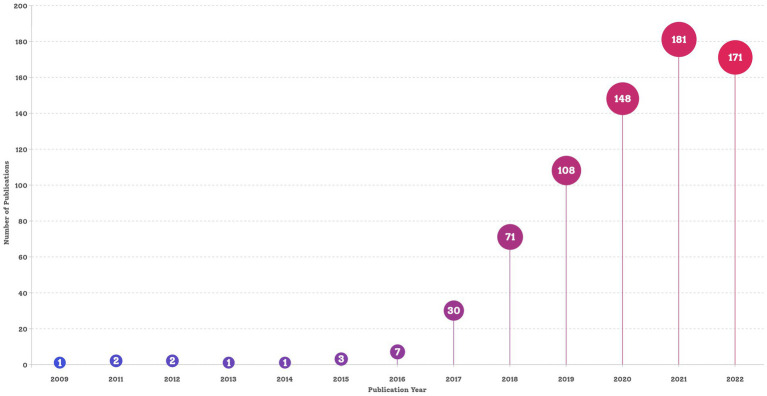
Trend of publications in the research field of *Candida auris*.

The final dataset contained 750 bibliographic records of English articles reporting on *C. auris* that were published before 28 March 2023. The search strategy and annual number of publications is shown in Figures 1, 2, respectively. The data for 2023 were not complete annual data; therefore, they are not displayed in Figure 2. The first report on *C. auris* was published in 2009. Over the past 13 years, the trajectory has exhibited two phases: an initial period (2009–2016), with only a small number of publications and a slow development pace, and a period of growth (2017–2022), with the number of annual publications peaking at 181 in 2021. The number of publications increased from 1 in 2009 to 171 in 2022, with an average of 2.1 in the period of 2009–2016 and 113.2 in the period of 2017–2022. The number of publications in the 3-year period of 2020–2022 reached 66.67% of the total number of publications (500/750). According to the number of citations, we selected 20 highly cited articles (with more than 100 citations) and summarized them in Supplementary Table S1. These articles covered different fields related to *C. auris*, including biofilm formation, identification methods, multi-drug resistance mechanisms, and nosocomial infections.

### 3.2 Countries

**Figure 3:**
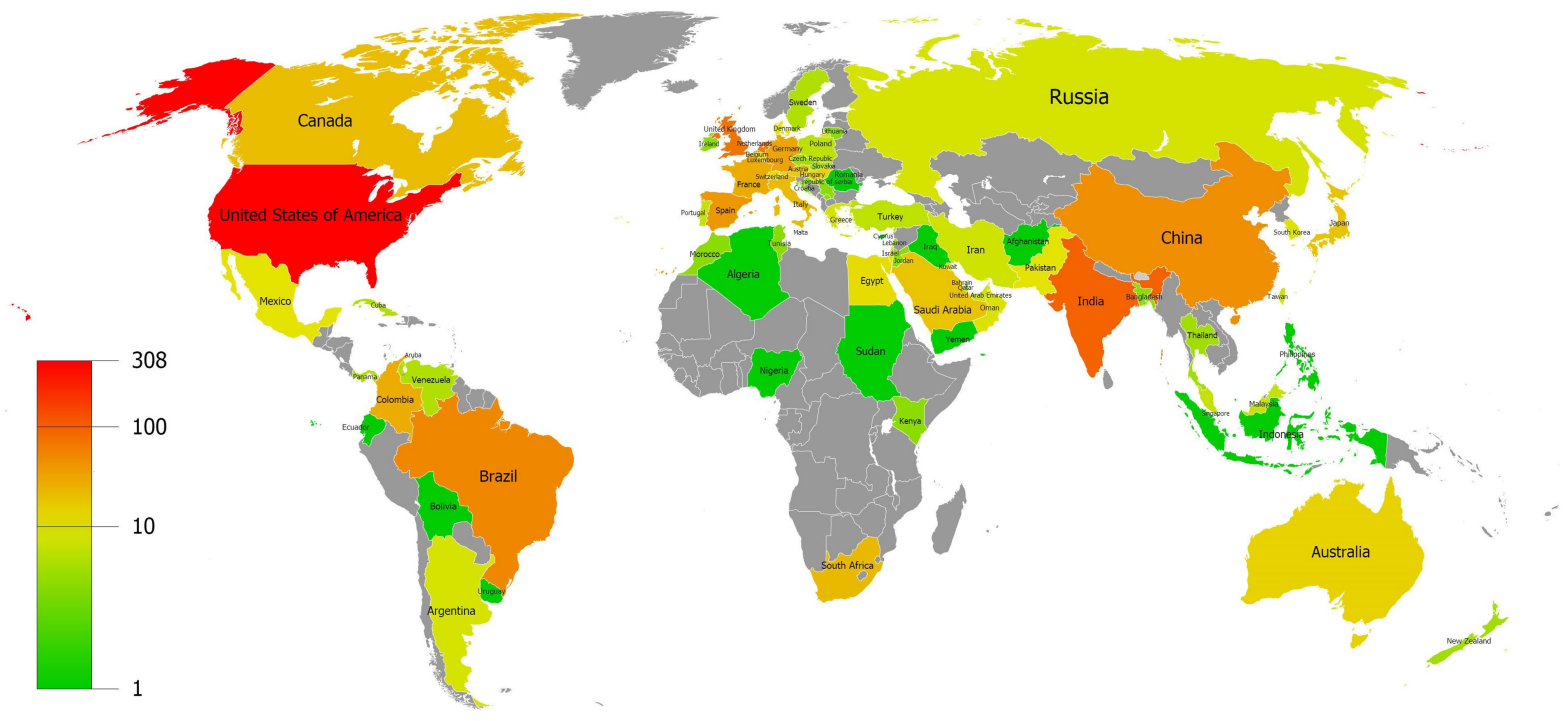
Distribution map of the countries/regions with different colors indicating different numbers of publications.

Our data revealed that 74 countries/regions have published relevant articles on *C. auris*. The global distribution of the countries/regions is shown in Figure 3, with different colors indicating different numbers of publications. The top 10 countries/regions based on the number of publications are listed in Table 1. The United States (308 counts, 9,380 citations) ranked first in terms of the number of publications and citations, followed by India (100 counts, 5,201 citations) and the United Kingdom (78 counts, 3,214 citations). Among the top 10 countries, the Netherlands had the highest average number of citations.

**Table 1:** Top 10 countries/regions in terms of the number of publications.

Rank	Country	Counts	Citations	Avg. citations	Avg. pub. year
1	USA	308	9,380	30	2020.10
2	India	100	5,201	52	2019.52
3	United Kingdom	78	3,214	41	2019.78
4	Netherlands	71	5,183	73	2019.30
5	Brazil	59	1801	31	2020.57
6	China	53	1,172	22	2020.38
7	Spain	46	1,499	33	2019.91
8	Germany	37	728	20	2020.29
9	Colombia	32	860	27	2019.84
10	France	32	643	20	2019.93

### 3.3 Journals

In total, 202 academic journals published articles related to *C. auris*, with the Journal of Fungi (63 articles) ranking first, followed by Antimicrobial Agents and Chemotherapy (58 articles) and Mycoses (30 articles). Table 2 shows the top 10 journals in terms of the number of publications. The top 10 journals published 294 articles, counting for 39.2% of the total. The Journal of Clinical Microbiology ranked first in terms of average citations, indicating that it was cited most frequently. Other than published amounts and citations, the Microbiology Spectrum had the latest Average Publication Year of 2021.42.

**Table 2:** Top 10 journals in terms of the number of publications.

Rank	Journal	IF2021	Counts	Citations	Avg. Citations	Avg. pub. year
1	Journal of Fungi		63	563	9	2020.95
2	Antimicrobial Agents and Chemotherapy		58	1711	30	2019.90
3	Mycoses		30	889	30	2019.73
4	Medical Mycology		25	400	16	2020.20
5	Journal of Clinical Microbiology		23	1,573	68	2018.30
6	Msphere		23	688	30	2019.83
7	Mbio		20	674	34	2020.50
8	Infection Control and Hospital Epidemiology		18	397	22	2019.75
9	Microbiology Spectrum		18	35	2	2021.42
10	Frontiers in Microbiology		16	186	12	2020.06

### 3.4 Institutions

As of 2023, 1,314 institutions have published in this field. Table 3 illustrates the top 10 institutions according to number of publications, 40% of which are in the Netherlands. The top five institutions that contributed the most to the field were the Centers for Disease Control and Prevention in the USA (48 counts), Delhi University (47 counts), Canisius Wilhelmina Hospital (32 counts), Radboud University Nijmegen (25 counts), and the University of the Witwatersrand (23 counts). Among the top 10 institutions, Radboud University Nijmegen had the highest average number of citations.

**Table 3:** Top 10 institutions in terms of the number of publications.

Rank	Institution	Counts	Citations	Avg. citations	Avg. pub. year
1	Centers for Disease and Prevention (United States)	48	2,891	60	2019.60
2	Delhi University (India)	47	4,414	94	2018.79
3	Canisius Wilhelmina Hospital (Netherlands)	32	2,967	93	2019.06
4	Radboud University Nijmegen (Netherlands)	25	2,573	103	2018.83
5	University of the Witwatersrand (South Africa)	23	298	13	2020.77
6	University of Exeter (United Kingdom)	21	232	11	2020.62
7	New York State Department of Health (United States)	21	783	37	2020.10
8	Westerdijk Fungal Biodiversity Institute (Netherlands)	17	686	40	2019.41
9	Canisius Wilhelmina Hospital (Netherlands)	17	1,051	62	2019.12
10	Postgrad Institute of Medical Education and Research (India)	17	488	29	2019.53

**Figure 4:**
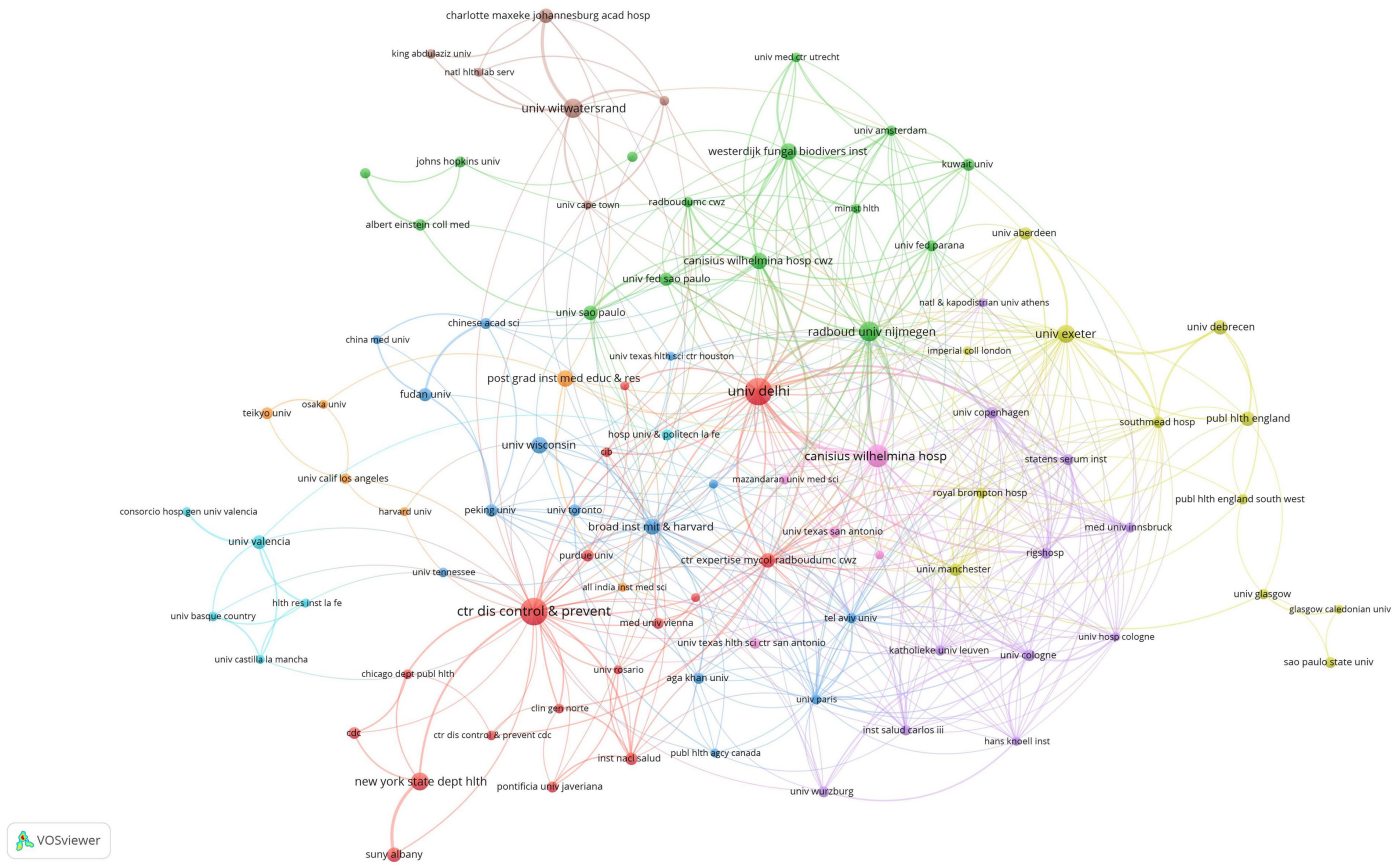
Institutional network map identified nodes and link lines, representing institutions and their collaborative relationships.

We set the threshold for the number of publications by institutions at 5 counts and identified 97 highly productive institutions from 1,314 institutions. We used VOSviewer to conduct a co-authorship analysis of 97 highly productive institutions, all of which were in a co-authorship network consisting of nine clusters. The generated institutional network map identifies the nodes and link lines that represent the institutions and their collaborative relationships. As shown in Figure 4, the red cluster was the largest, consisting of 17 institutions. Centres for Disease and Prevention work with 39 highly productive institutions and ranked first. This was closely followed by the Canisius Wilhelmina Hospital, which works with 36 highly productive institutions, and Delhi University and Radboud University Nijmegen, which work with 34 highly productive institutions.

### 3.5 Authors

In total, 4,105 authors have published in this field since 2009. Table 4 lists the top 10 authors in terms of number of publications. Jacques F. Meis has published the most articles (48) and is ranked first, followed by Anuradha Chowdhary (40) and Shawn R. Lockhart (32). Anuradha Chowdhary had the highest average citations (3808).

**Table 4:** Top 10 authors in terms of the number of publications.

Rank	Author	Counts	Citations	Avg. citations	Avg. pub. year
1	Jacques F. Meis	48	3,692	77	2019.30
2	Anuradha Chowdhary	40	3,808	95	2018.95
3	Shawn R. Lockhart	32	2,285	71	2019.06
4	Elizabeth L. Berkow	22	1999	91	2018.86
5	Anastasia P. Litvintseva	20	1992	100	2019.25
6	Arunaloke Chakrabarti	19	438	23	2019.89
7	Andrew M. Borman	18	774	43	2019.89
8	Snigdha Vallabhaneni	18	1,436	80	2019.17
9	Kaitlin Forsberg	17	647	38	2020.24
10	Sudha Chaturvedi	16	545	34	2020.38

**Figure 5:**
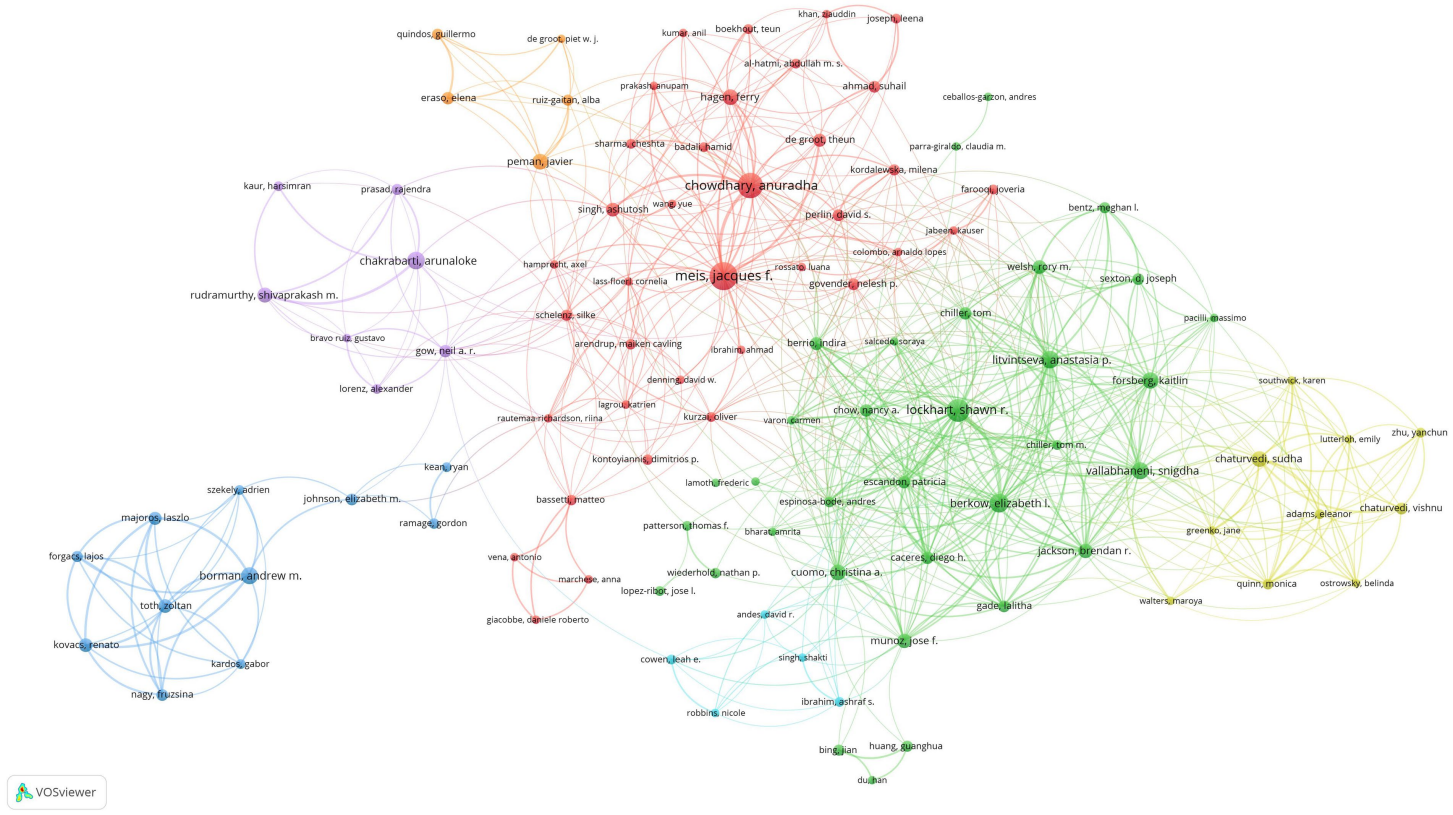
Co-authorship network map identified nodes and link lines, representing authors and their collaborative relationships.

**Figure 6:**
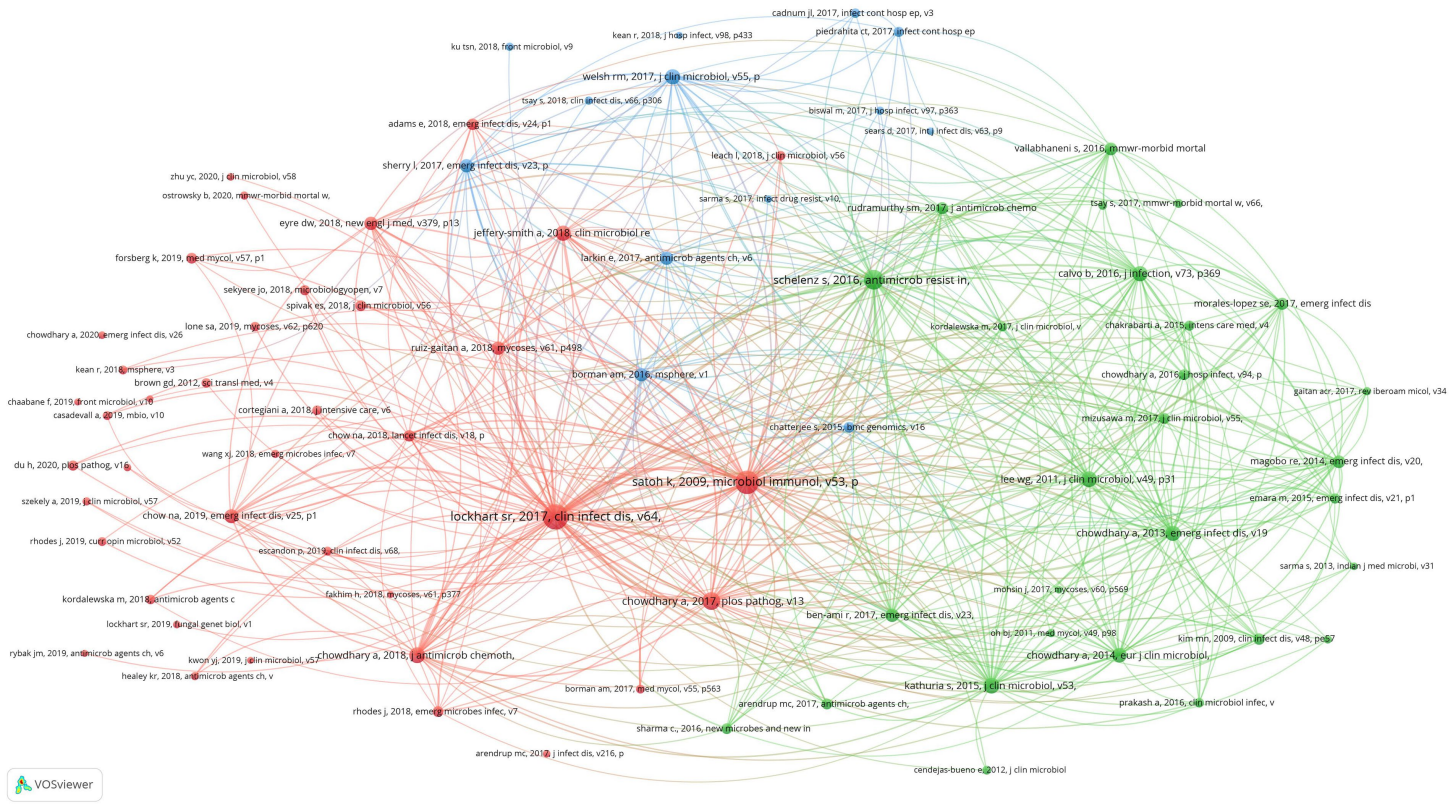
Co-citation network map identified nodes and link lines, representing references and their co-citation relationships.

We set the threshold for the number of publications by authors at 5 counts and identified 127 highly productive authors from 4,105 authors. We used VOSviewer to conduct a co-authorship analysis on 127 highly productive authors, 107 of whom formed the largest co-authorship network, consisting of 7 clusters. The generated co-authorship network map identifies nodes and link lines representing authors and their collaborative relationships. Approximately 85% of the highly productive authors are in the co-authorship network, indicating that collaboration among highly productive authors is considerably active. According to the statistics on citation and publication amounts, Jacques F. Meis has published the highest number of related articles, while Anastasia P. Litvintseva’s articles have the highest average citation rate. This result was indicated in Table 4.Furthermore, as shown in Figure 5, Meis worked with 46 highly productive authors and ranked first. He is closely followed by Shawn R. Lockhart, who worked with 44 highly productive authors, and Elizabeth L. Berkow, who worked with 39 highly productive authors. Jacques F. Meis and Anuradha Chowdhary had close partnerships and cooperated in 21 collaborations, ranking first in terms of number of collaborations among highly productive authors. Borman formed a close partnership in the relatively separate blue cluster shown at the bottom left of Figure 6. The authors in this cluster have 11 collaborations with each other, and most articles should have been published together. This may indicate inadequate collaboration between them and other top authors.

### References

3.6

Scientific studies are based on the research of predecessors, and we can better understand the knowledge base of a certain research field by analyzing references. In this study, 15,892 references were cited in 750 research articles on *C. auris*, appearing 30,728 times, with an average of 41 references per article. Overall, 80 references have been cited more than 30 times. We used VOSviewer to conduct a co-citation analysis of the 80 references and generate visual maps.

### 3.7 Keywords

In total, 2,465 keywords occurred in 750 articles. We set the threshold for high-frequency keywords to 10 counts and identified 98 high-frequency keywords. Excluding the search keyword *C. auris*, the top 5 keywords were resistance (201 counts), *C. albicans* (151 counts), antifungal susceptibility (102 counts), infections (94 counts), and identification (90 counts).

**Figure 7:**
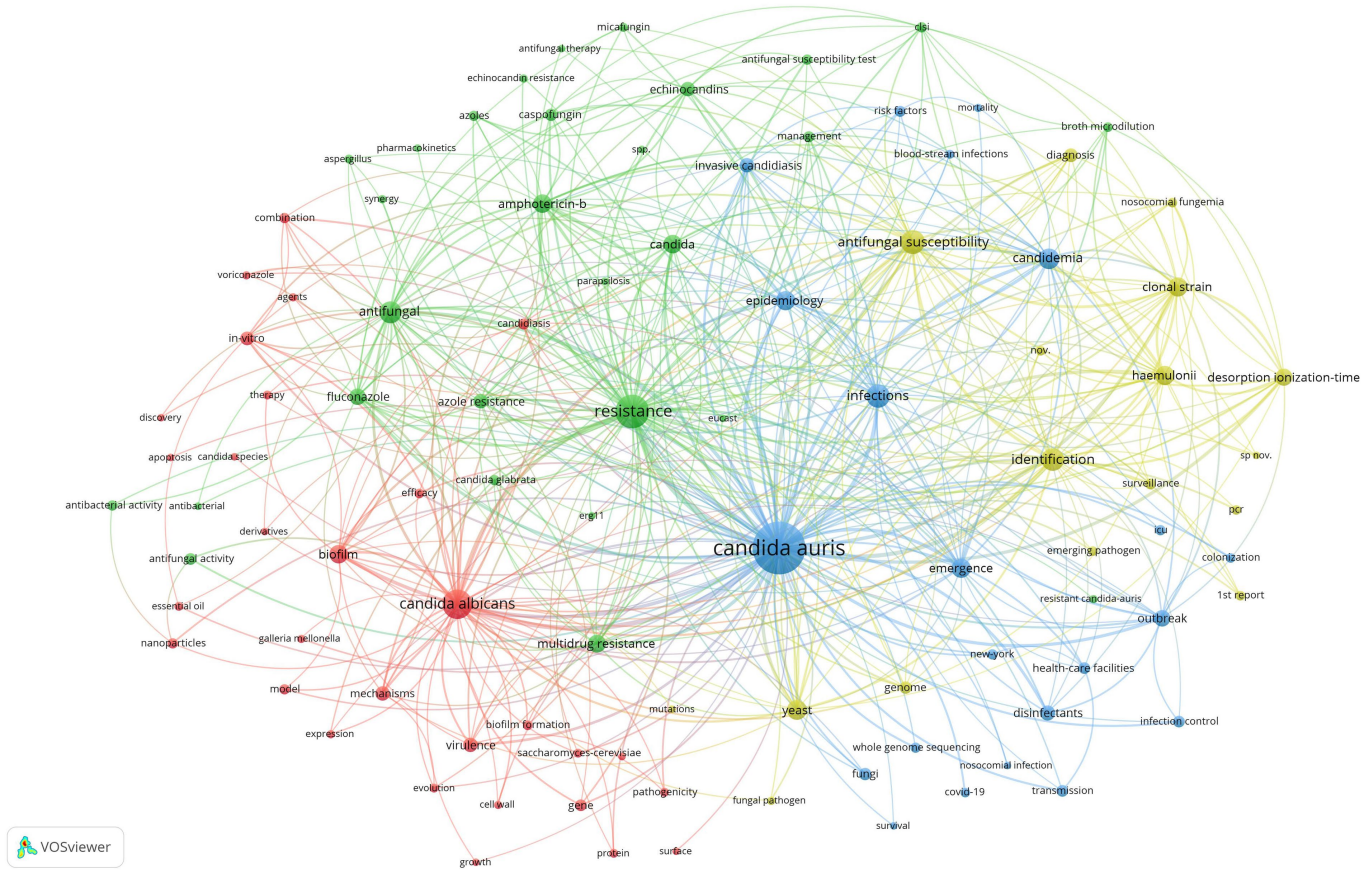
Co-contribution network map identified nodes and link lines, representing keywords and their co-occurrence relationships.

Co-occurrence analysis was performed on the 98 high-frequency keywords, and a co-contribution network map of the high-frequency keywords was constructed. The co-occurrence analysis of the 98 high-frequency keywords formed 4 clusters, which are represented by different colors in Figure 7. The red cluster comprised 30 keywords and formed the largest cluster. Cluster1 (red), Cluster2 (green), Cluster3 (blue), and Cluster4 (yellow) focus on research topics 1, 2, 3, and 4, respectively.

## 4 Discussion

This is the first bibliometric study of *C. auris* aimed at analyzing important information, such as the number of publications, publication region, authors, references, and keywords, using VOSviewer (version 1.6.19) software for visualization.

### 4.1 Trends in publications

Since the discovery of *C. auris* in 2009, 750 articles on *C. auris* have been published. Since 2020, the number of studies on *C. auris* has increased exponentially. Article published during the 2020–2022 period accounted for 66.67% of the total publications, indicating that this particular research field has become more attractive to researchers.

The United States published the most articles on *C. auris*, followed by India, the United Kingdom, and the Netherlands. According to the average number of publication years, the average value for the United States is higher than that of other countries. Considering that the number of publications in the United States was also ranked first, this indicates that the United States has published more recently with regards to *C. auris*. According to statistics, the Journal of Clinical Microbiology had the highest average number of citations and a relatively early publication year (average, 2018.3). These articles mainly focused on new progress in the identification of *C. auris*. This is possibly due to the lack of appropriate laboratory identification technology for *C. auris*, which is prone to identification errors (Mizusawa et al., 2017; Das et al., 2021). In contrast, Mbio published more recent articles and had a higher single citation rate. The content of the published articles mainly dealt with the evolutionary process of *C. auris* (Casadevall et al., 2019, 2021; Chow et al., 2020; Yadav et al., 2022) and the mechanism of drug resistance (Kim et al., 2019; Rybak et al., 2020; Carolus et al., 2021), which is in line with current research hotspots.

### 4.2 International Collaboration

The co-authorship analysis of the research institutions revealed that the United States, the Netherlands, the United Kingdom, and India accounted for the top 10 institutions in terms of publication numbers, while Centers for Disease and Prevention, Canisius Wilhelmina Hospital, Delhi University, and Radboud University Nijmegen were the four institutions that generally worked with other highly productive institutions the most. This shows that these four institutions are currently the centers of cooperation among global institutions in the *C. auris* research field.

Further analysis of the highly productive authors revealed that half of the top 10 authors, in terms of publication number, were from the Centers for Disease Control and Prevention, with a significant collaboration among these authors. Since the COVID-19 epidemic, *C. auris* outbreaks have occurred in ICUs and hospitals treating COVID-19 patients and in various environmental settings in hospitals (Sharma and Kadosh, 2023). This has attracted the attention of the Centers for Disease Control and Prevention (CDC) and is consistent with the fact that many of its most productive authors come from the CDC. For instance, Anastasia P. Litvintseva has published 20 articles with approximate 2,000 citations, and her main research interests include the origin of *C. auris* (Jackson et al., 2019; Chow et al., 2020), nosocomial outbreaks, molecular epidemiology of *C. auris* (Wilson et al., 2018; Armstrong et al., 2019; Escandon et al., 2019; Proctor et al., 2021), novel identification modes of *C. auris* (Sexton et al., 2018), and biological characteristics of *C. auris* (Munoz et al., 2018). Her studies cover the latest research hotspots on *C. auris* and establish an important position in the field of *C. auris* research.

### 4.3 Research hotspots

The high-frequency keywords derived from the bibliometric and visual analysis of *C. auris* articles can be roughly classified into four research directions: pathogenicity, drug resistance, epidemiological characteristics, and rapid and precise identification technology.

#### 4.3.1 Pathogenicity research

As a critical human pathogen, researchers are still drawing attention to the pathogenicity and virulence of *C. auris*. *Candida auris* has a strong biofilm-forming ability, which not only enhances its efflux ability against fungal drugs (Bravo Ruiz and Lorenz, 2021), but also enhances its resistance to antifungal drugs, such as azoles, echinocandins, and polyenes (Kean and Ramage, 2019). In addition, *C. auris* can cause nosocomial infections through skin colonization or contamination of the surfaces of medical facilities (Kumar et al., 2019), and its ability to form biofilms has resulted in a certain degree of resistance to a variety of hospital disinfectants (Ledwoch and Maillard, 2018). Therefore, the formation mechanisms and elimination methods of *C. auris* biofilms have emerged as current research hotspots. The ability of *C. albicans* to form biofilms is much stronger than that of other *Candida* species under typical experimental conditions; however, Nett et al. found that the biofilm produced by *C. auris* in a simulated axillary skin model was 10 times higher than that of *C. albicans*. This also explains the difficulty in eradicating *C. auris* in hospital settings (Horton et al., 2020). Hu et al. evaluated the killing effects of a variety of chemical disinfectants, including three hand hygiene products, ultraviolet radiation, and ozone, on *C. auris* (Fu et al., 2020); they found that *C. auris* has strong resistance to quaternary ammonium salts, ultraviolet radiation, and ozone disinfection and the disinfection time must be extended to effectively inhibit its growth. However, their evaluation process did not consider the killing ability of these sterilization methods on the biofilm of *C. auris* (Gowri et al., 2020). Therefore, methods for inhibiting or eradicating the biofilms of *C. auris* formed in skin models will be an important and valuable research topic in the future.

An experiment conducted using mice as an animal model revealed that the virulence of *C. auris* was comparable to that of *C. albicans* (Fakhim et al., 2018); when fruit flies were used as the host, the lethality rate of *C. auris* was even higher than that of *C. albicans* (Wurster et al., 2019), confirming the strong pathogenicity of *C. auris*. Compared to *C. albicans*, *C. auris* colonizes the skin more deeply, resulting in invasive candidaemia, and IL-17 plays an important role in skin and subcutaneous tissue infections (Huang et al., 2021). Another study on host immune responses to *C. auris* revealed that *C. auris* has a strong immune evasion ability (Johnson et al., 2018) and further studies may show that this ability may be derived from PD-1 (Wu et al., 2019), but the specific mechanism must be studied further. In short, *C. auris* has strong virulence and immune evasion ability, and the study of the underlying mechanisms may have preventive and therapeutic significance.

#### 4.3.2 Drug resistance of *Candida auris*

The multidrug resistance of *Candida* (Lockhart et al., 2017) is one of the most important characteristics that has attracted attention, and the resistance mechanism of the *Candida* species has always been a key focus of research. Recent studies have reported that the resistance of *C. auris* to echinocandins is related to the hotspot mutation of FKS1 (Chowdhary et al., 2018; Kordalewska et al., 2018); its resistance to azoles is generally related to ERG11 mutations, including K143R of F126L and Y132F (Chowdhary et al., 2018; Chow et al., 2020). Recently, Chaturvedi et al. found that the lack of FUR11 is a novel flucytosine resistance mechanism apart from FCY1, FUR1, and ADE17 mutations; however, in an amphotericin B (AmB) resistance mechanism verification study, they did not find any statistically significant nonsynonymous variants in sensitive or drug-resistant strains. Therefore, the resistance mechanism of amphotericin remains unclear and is also a direction for further research. For multidrug-resistant *C. auris*, synergistic effects between different antifungal drugs and between antibacterial drugs and antifungal drugs are important research topics for the treatment of *C. auris* infections, such as voriconazole and micafungin (100%) and flucytosine and amphotericin (100%). Further studies are required to validate these experimental data (Aghaei Gharehbolagh et al., 2021). Novel antifungal drugs, such as rezafungin, ibrexafungerp, olorofim, and fosmanogepix, have demonstrated efficacy against *C. auris* in *in vitro* studies (Lamoth et al., 2022; Sobel et al., 2022); however, their clinical efficacy needs to be evaluated in future clinical studies. In addition, several studies have determined the critical roles of nanoparticles in inhibiting the growth and biofilm formation of *C. auris*. Although these conclusions have not been tested in a clinical trial, nanoparticles have displayed great potential in treating *C. auris* infection and decolonization (Fayed et al., 2021; Kamli et al., 2021a,b; AlJindan and AlEraky, 2022; Bandara and Samaranayake, 2022).

#### 4.3.3 Epidemiology studies of nosocomial transmission of *Candida auris*

Since the first strain of *C. auris* was isolated in 2009, this pathogen has posed enormous threats to humans due to several of its pathogenic properties. In 2011, the first three clinical cases of nosocomial fungaemia caused by *C. auris* were reported in South Korea; two of these patients died due to septic shock and multiple-organ failure and had received fluconazole therapy. Further, AmB displayed therapeutic failure. Besides the fact that it is multi-drug resistant, its ability to persist, easily transmit, and its highly proliferative clonal lifecycle endows this fungus with an expanding range of nosocomial infections globally (Lee et al., 2011; Schelenz et al., 2016; Vallabhaneni et al., 2017). In the United States, *C. auris* strains are associated with nosocomial outbreaks, and the excess healthcare costs have increased from $35,000 to $68,000 (Suleyman and Alangaden, 2021). From 2013 to 2018, 18 studies involving more than 200 patients reported nosocomial infections in more than 10 countries (Dahiya et al., 2020). Furthermore, according to several clinical descriptions, invasive candidiasis caused by *C. auris* is easily misdiagnosed as *Saccharomyces cerevisiae, C. famata* or other *Candida* species (Lee et al., 2011; Schelenz et al., 2016). Thus, precise and efficient identification is critical for controlling *C. auris*-mediated nosocomial transmission.

Since the onset of the COVID-19 epidemic, a large number of patients with severe COVID-19 pneumonia have been admitted to the ICU, and many cases of *C. auris* coinfection have emerged (Chowdhary et al., 2020; Janniger and Kapila, 2021; Kariyawasam et al., 2022; Thoma et al., 2022). In India, two-thirds of COVID-19 deaths were caused by concomitant *C. auris* infections (Chowdhary et al., 2020), and similar scenarios were observed in Mexico and Israel. From 2021 to 2022, the *C. auris* incidence rate increased more than 30-fold, corresponding with the outbreak of the COVID-19 pandemic in Israel. Further, more than 23% of patients suffer from coinfections with these two pathogenic factors; among these co-infected patients, more than 70% require mechanical ventilation (Biran et al., 2023). One study reported 12 patients infected with both *C. auris* and SARS-CoV-2 in an intensive care unit (ICU) in Mexico in 2020. Fungal strains were isolated from blood or urine samples, or both. The mortality rate is generally >80% (Villanueva-Lozano et al., 2021). Unlike the above-mentioned national studies, one study demonstrated an outbreak of *C. auris* alongside COVID-19 in a specialty care unit in Florida from July to August 2020. Researchers have indicated that the spread of COVID-19 has significantly enhanced the nosocomial infection of *C. auris*, in contrast to the situation in 2017. Separate medical care for mono-infections is highly recommended to avoid crossing infection and nosocomial transmission (Prestel et al., 2021).

Although there were many cases indicating a strong correlation between COVID-19 and *C. auris* infection, as mentioned above, this critical fungal pathogen caused serious nosocomial infection outbreaks prior to the presence of SARS-CoV-2. From the scientific article retrieval, studies have demonstrated that the mortality rate caused by *C. auris* infection was 30–60% during the pre-COVID-19 era (Lee et al., 2011; Chowdhary et al., 2013, 2014; Lockhart et al., 2017; Ruiz-Gaitan et al., 2018). During the outbreak of nosocomial infections, several predisposing factors of *C. auris* colonization and candidemia were monitored. Javier Pemán and his team pointed that polytrauma, cardiovascular disease, and cancer were the most common physiological conditions in *C. auris* infected patients. Moreover, the indwelling central venous catheters (CVC), parenteral nutrition, and mechanical ventilation act as the critical predictors of candidemia. These data were collected from a 12-month prospective, case–control study that included a total of 228 patients (Ruiz-Gaitan et al., 2019). Moreover, administration of immunosuppressors, antifungal therapies and long-term ICU accommodation also increase the risk of *C. auris* colonization (Eyre et al., 2018; Ruiz-Gaitan et al., 2018, 2019). Furthermore, the risk factors for ICU outbreaks include non-compliance with personal protective equipment (PPE) use, hand hygiene, PPE shortages, and excessive antibiotic use (Thoma et al., 2022). The multi-drug resistance and high fatality rate of *C. auris* pose great challenges in the treatment of patients. Therefore, the focus of further research will be to prevent nosocomial transmission through early detection of nosocomial epidemics using rapid and effective identification, summary analysis of infection cases, proposals for more effective treatment methods based on new antifungal drugs, and the implementation of more effective combinations of disinfection methods and infection control programs.

#### 4.3.4 Rapid and precise identification technology of *Candida auris*

Pathogen identification methods should be highly accurate, low-cost and time-efficient. As mentioned above, *C. auris* is spreading globally and has been identified in various countries. Genetically, *C. haemulonii*, *C. auris*, *C. pseudohaemulonii*, and *C. duobushaemulonii* are closely related yeast pathogens and display highly multidrug resistant abilities. Thus, low-cost and precise identification methods are required to distinguish these different pathogens.

Since the first identification of *C. auris* in 2009, it has attracted widespread attention due to the difficulty in distinguishing it from *C. haemulonii* using traditional methods and its high level of resistance to azoles (Satoh et al., 2009; Kathuria et al., 2015; Kumar et al., 2016). There are five clades of *C. auris* with tens of thousands of single-nucleotide polymorphisms among clades (Wilson et al., 2018; Spruijtenburg et al., 2022), which result in differences in the drug resistance of each clade (Chowdhary et al., 2018). As these five clades of *C. auris* are widespread (Waters et al., 2023), it has become important to quickly distinguish between the five clades and develop different response plans according to the characteristics of each clade to control clinical infection. From 2017, there were three studies have demonstrated the Polymerase Chain Reaction (PCR) based method enable to identify the *C. auris* and phylogenetic relatives (Kordalewska et al., 2017; Arastehfar et al., 2018, 2019). Beside the aforementioned molecular method, Elizabeth M Johnson has established a novel and expeditious method for identifying the *C. auris* isolates from patient samples, which was named as the CHROMagarTM Candida Plus (Borman et al., 2021). Besides above mentioned methods, the MALDI-TOF MS and ITS sequencing can only be used to identify *C. auris* species but cannot distinguish between the five clades. Although whole genome sequencing can be used to distinguish between the five clades, it is time-consuming and expensive. A recent study by Jacques F. Meis et al. identified *C. auris* at the level of different clades through high-resolution mass spectrometry (Jamalian et al., 2023) and this provides us with new tools for faster and more accurate identification for a rapid response to nosocomial *C. auris* infection and to trace the source of the infection. In addition, YANG et al. applied recombinase-aided amplification combined with lateral flow strips (RAA-LFS) to detect *C. auris* and successfully developed a rapid and effective method for this purpose (Zhang et al., 2023). In conclusion, *C. auris* identification methods that are faster, cheaper, and have higher resolutions remain the focus of future research.

### 4.4 Limitations

Like other bibliometric studies, this study also has some limitations. First, to conduct bibliometric analysis, bibliographic data must be extracted from database such as WOS, Scopus, Dimensions, and PubMed. Due to the different formats of bibliographic data, current bibliometric research can only select one database for analysis. In this study, we only used WOS for data retrieval. WOS is the largest comprehensive academic information resource that provides large coverage of over 20,000 peer-reviewed journals. Consequently, many researchers have used WOS to conduct bibliometric studies on viral diseases with similar objectives to the present study. However, WOS cannot do all the related articles, which may also cause bias in the results. Second, we only used VOSviewer software tool to achieve our analyses. Through article retrieval in WOS database about the bibliometric analyses, both of VOSviewer and CiteSpace software enable to achieve bibliometric analyses, and they share lots of functionalities that include Co-Occurrence-, Co-Citation- and Co-Authorship- analyses. To compare with CiteSpace, VOSviewer is user-friendly and CiteSpace displays advantages in the evaluative analysis of network visualization. Moreover, the statistical analyses are relying on the authorship of researchers and institutions via VOSviewer software tool, the outcome of data may be biased by the unstandardized manner or same name authors.

## 5 Conclusion

Using bibliometrics and visualization analyses, this study comprehensively evaluated the global research trends and hotspots in the research field of *C. auris* and predicted future research directions. Close cooperation between institutions has occurred over the past decade, and the number of publications has increased rapidly since 2009. The United States, Netherlands, United Kingdom, and India have contributed the most to this area of research. In addition to the identification, drug resistance, and treatment of *C. auris* infections, in-hospital outbreak control at the source should be considered an important research focus.

## Data availability statement

The raw data supporting the conclusions of this article will be made available by the authors, without undue reservation.

## Author contributions

QW: Formal analysis, Funding acquisition, Supervision, Validation, Writing – original draft, Writing – review & editing, Methodology. SC: Conceptualization, Investigation, Supervision, Writing – review & editing. YW: Data curation, Formal analysis, Resources, Visualization, Writing – review & editing. FL: Writing – review & editing. JC: Writing – review & editing. WD: Writing – original draft, Resources, Validation, Visualization, Writing – review & editing. HK: Conceptualization, Supervision, Writing – review & editing. ZW: Formal analysis, Methodology, Supervision, Writing – original draft.
