# Supplementary material for: Global characteristics and trends in research on Candida auris
Source: Front Microbiol. 2023 Dec 6;14:1287003. doi: 10.3389/fmicb.2023.1287003 (PMC10731253; doi:10.3389/fmicb.2023.1287003)
Supplement: Supplementary file 1 [file Table_1.docx]

**Supplemental Table 1:** High cited articles on *C. auris*

| **Articles** | **Number of Citations** |
| --- | --- |
| lockhart sr, 2017, clin infect dis, v64, p134, doi 10.1093/cid/ciw691 | 375 |
| satoh k, 2009, microbiol immunol, v53, p41, doi 10.1111/j.1348-0421.2008.00083.x | 319 |
| schelenz s, 2016, antimicrob resist in, v5, doi 10.1186/s13756-016-0132-5 | 220 |
| chowdhary a, 2017, plos pathog, v13, doi 10.1371/journal.ppat.1006290 | 178 |
| kathuria s, 2015, j clin microbiol, v53, p1823, doi 10.1128/jcm.00367-15 | 151 |
| lee wg, 2011, j clin microbiol, v49, p3139, doi 10.1128/jcm.00319-11 | 146 |
| chowdhary a, 2018, j antimicrob chemoth, v73, p891, doi 10.1093/jac/dkx480 | 144 |
| calvo b, 2016, j infection, v73, p369, doi 10.1016/j.jinf.2016.07.008 | 139 |
| jeffery-smith a, 2018, clin microbiol rev, v31, doi 10.1128/cmr.00029-17 | 139 |
| chowdhary a, 2013, emerg infect dis, v19, p1670, doi 10.3201/eid1910.130393 | 133 |
| welsh rm, 2017, j clin microbiol, v55, p2996, doi [10.1128/jcm.00921-17 10.1128/jcm.00921-17] | 133 |
| chowdhary a, 2014, eur j clin microbiol, v33, p919, doi 10.1007/s10096-013-2027-1 | 126 |
| chow na, 2019, emerg infect dis, v25, p1780, doi 10.3201/eid2509.190686 | 109 |
| eyre dw, 2018, new engl j med, v379, p1322, doi 10.1056/nejmoa1714373 | 106 |
| ruiz-gaitan a, 2018, mycoses, v61, p498, doi 10.1111/myc.12781 | 105 |
| sherry l, 2017, emerg infect dis, v23, p328, doi 10.3201/eid2302.161320 | 105 |
| larkin e, 2017, antimicrob agents ch, v61, doi [10.1128/aac.02396-16 10.1128/aac.02396-16] | 102 |
| ben-ami r, 2017, emerg infect dis, v23, p195, doi 10.3201/eid2302.161486 | 101 |
| borman am, 2016, msphere, v1, doi 10.1128/msphere.00189-16 | 100 |
